# Disease Modeling with Kidney Organoids

**DOI:** 10.3390/mi13091384

**Published:** 2022-08-25

**Authors:** Sophie Karp, Martin R Pollak, Balajikarthick Subramanian

**Affiliations:** Division of Nephrology, Department of Medicine, Beth Israel Deaconess Medical Center, Harvard Medical School, Boston, MA 02215, USA

**Keywords:** organoids, kidney, development, metanephros, ureteric, tubule, chip, nephrology, disease-modeling

## Abstract

Kidney diseases often lack optimal treatments, causing millions of deaths each year. Thus, developing appropriate model systems to study human kidney disease is of utmost importance. Some of the most promising human kidney models are organoids or small organ-resembling tissue collectives, derived from human-induced pluripotent stem cells (hiPSCs). However, they are more akin to a first-trimester fetal kidney than an adult kidney. Therefore, new strategies are needed to advance their maturity. They have great potential for disease modeling and eventually auxiliary therapy if they can reach the maturity of an adult kidney. In this review, we will discuss the current state of kidney organoids in terms of their similarity to the human kidney and use as a disease modeling system thus far. We will then discuss potential pathways to advance the maturity of kidney organoids to match an adult kidney for more accurate human disease modeling.

## 1. Introduction

Until recently, human diseases have been modeled in the following two ways: with animals or with two-dimensional cell culture. Animal models allow for the study of disease pathways and drug development in whole organisms, but they do not account for human genetic and anatomical makeup. On the other hand, human cell culture allows scientists to study human-specific disease mechanisms and evaluate treatments to a great and precise molecular depth. However, cell culture does not recapitulate an organ’s complex three-dimensional structure and physiology.

Human organoids are simplified versions of human organs, with realistic three-dimensional microanatomy and organ-level functions (e.g., tear production in lacteal organoids and hair growth in skin organoids) [1,2]. Through self-organization processes, they are derived in vitro from tissue cells or pluripotent stem cells. Induced pluripotent stem cells (iPSCs), invented in 2008 by Takahashi and Yamanaka, are the most popular cells for organoid formation [3]. This is due to several compelling advantages that iPSCs offer. Firstly, they provide an unlimited renewable cellular source and can differentiate into multiple cell types. More importantly, they represent the underlying genetic composition of patients. Thus, patient-derived human iPSCs (hiPSCs) may be used to study disease via organoids at the individual level, allowing for advancements in personalized medicine. To date, organoid models of various human organs, such as the heart and the brain, have been used to study disease. For this review, we will focus on kidney organoids and discuss how they may be used to model the human kidney and its associated diseases, screen for drug toxicity, and perhaps supplement kidney function.

Kidney disease affects 10% of the world’s population [4]. Despite identifying the underlying genetic cause for many forms of kidney diseases, there are no optimal therapies available to manage them. This unfortunate situation reflects the lack of in vitro model systems that recapitulate human kidney disease for targeted therapeutic development. Kidney organoids offer a human-based, personalizable, 3-D research platform to investigate genetic kidney diseases, kidney injury, and drug toxicity. Along with its capacity to serve as a modeling system, a kidney organoid possesses the potential to advance transplantation medicine. Thus, to maximize its potential in both these fields, the kidney organoid must resemble adult human kidney structure and function. Here, we will review the current state of the pluripotent cell-derived human kidney organoid, its potential uses, and areas for improvement.

## 2. The Human Kidney vs. Kidney Organoids

HiPSCs serve as proxies for embryonic PSCs. Thus, forming a kidney organoid from hiPSCs entails replicating human embryonic kidney development. Below, we will explore how the kidney organoid is developed and compare it against the human kidney.

### 2.1. Human Kidney Development

**Figure 1:**
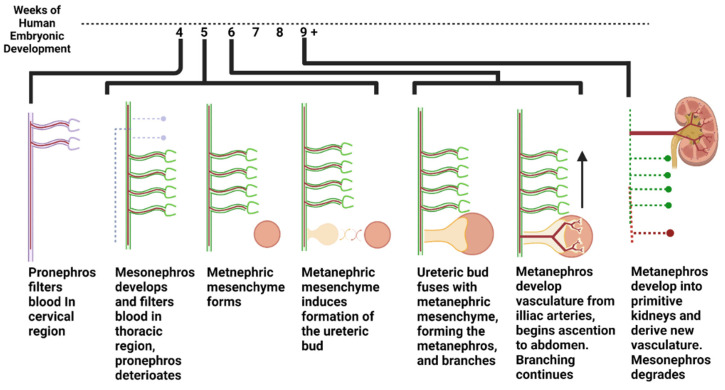
Development of the human urinary system from weeks 4 to 9 in womb.

Blood is filtered in three distinct phases throughout embryonic development. In the first phase, a structure termed the pronephros develops in the 4-week-old human embryo and processes blood in the cervical region until around week 5 [5]. Next, the mesonephros forms in the thoracic region of the embryo and filters the blood from the start of week 5 through about week 10 of embryonic development. While the mesonephros is filtering blood, the final filtration system, which will eventually become the adult kidney, begins to form (Figure 1). It forms in the following two parts: the metanephric mesenchyme and the ureteric bud. The metanephric mesenchyme forms all parts of the nephron except the collecting duct, while the ureteric bud forms the collecting duct. These structures go through a reciprocal induction cycle, wherein the metanephric mesenchyme and ureteric bud stimulate the growth of one another and fuse, forming a structure called the metanephros. The newly formed metanephroi filter blood from the iliac branches down to the pelvic region. At the same time, the ureteric bud bifurcates and continues to branch to form many collecting ducts within the kidney [6,7]. Subsequently, these fused metanephric structures ascend into the abdomen region of the embryo as it develops and forge blood supply from the primitive aorta. These processes are summarized in Figure 1. It is important to note that metanephric mesenchyme and ureteric bud cells are primitive, and thus maintain multipotent potential, whereas adult differentiated kidney cells are committed to their particular lineage [8].

### 2.2. Protocols to Generate Kidney Organoids

Kidney organoids can be formed from tissue-derived differentiated cells or pluripotent stem cells. To develop a kidney organoid from tissue-derived cells, one may arrange the differentiated adult cells in three-dimensional space ex vivo to mimic human organ architecture [9]. Organoid protocols for tissue-derived differentiated cells are covered elsewhere. In this review, we will focus on stem cell-derived kidney organoids. To create these systems, scientists may culture pluripotent stem cells in the presence of kidney-specific endogenous morphogens and extracellular components. Stem cells can then self-assemble into kidney-like structures, mimicking embryonic kidney development. Organs, such as the intestine, harbor endogenous stem cell populations in the adult tissue with which scientists may create organoids [10]. However, since there has been no clear evidence that an adult human kidney contains a stem cell niche, stem cell-derived kidney organoids must be made from pluripotent stem cells (either embryonic or hiPSC) [11].

Stem cell-derived kidney organoids may be classified into the following two categories: nephron progenitor (NP) organoids and ureteric bud (UB) organoids. Both the types of organoids have been well established as disease models. NP organoids resemble the metanephric mesenchyme, which contains multipotent NP cells. In fact, a single metanephric mesenchymal cell can give rise to all epithelial cells of the nephron, excluding the collecting duct [8]. Methodologies for generating hiPSC-derived NP organoids include 2D culture with subsequent aggregation in a porous transwell plate (e.g., Takasato et al. (2016), Morizane et al. (2015)) or 3D culture in a hydrogel (e.g., Freedman et al. (2015)) [12,13,14]. These organoids develop glomerular and tubular structures. NP organoids derived from two of the most popular NP protocols by Takasato and Morizane were compared in an extensive omics analysis by Wu et al. (2018) [15]. They found that while the Takasato protocol generates about 11% podocyte-like cells and 21% off-target cells per organoid, the Morizan protocol generates about 28.5% podocyte-like cells and 14.3% off-target cells [15]. Takasato’s organoids also appear to develop a small amount of UB-like regions, but predominantly imitate the metanephric mesenchyme, thus classifying them as NP organoids [12]. Furthermore, Morizane et al. (2015) and Takasato et al. (2016)’s protocols lack an extracellular hydrogel environment that is present in that of Freedman et al.’s (2015) work [14]. Garreta et al. (2019) argue that the presence of a hydrogel in organoid formation improves kidney structure formation and enhances the production of early IM markers, posterior IM markers, and anterior IM markers [16].

**Figure 2:**
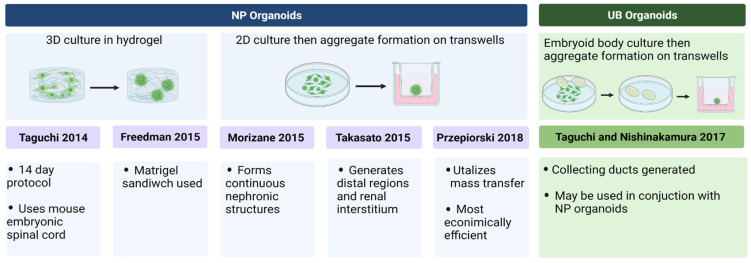
Summary of the most popular organoid formation protocols.

In contrast to NP organoids, UB organoids imitate the ureteric bud, which gives rise to the collecting duct system. Methods for generating UB organoids have been more recently developed and include embryoid body cultivation and subsequent aggregation to low-adherent wells [17]. These organoids have tubular and collecting duct structures. Finally, NP and UB organoids have also been combined to generate higher-order co-culture structures to recapitulate adult human kidney phenotypes [17]. The most popular protocols for kidney organoid formation are summarized in Figure 2 below [12,13,14,17,18,19].

### 2.3. How They Stack Up: Kidney Organoids vs. Human Kidnies

The protocols described above primarily involve the self-assemblage of hiPSCs into organoids. They mimic fetal conditions to induce hiPSCs to differentiate into kidney-specific lineages and form kidney-specific structures. The first fetal condition they replicate is primitive streak signaling. The primitive streak is a section of the embryo that develops before the three germ layers separate. Most protocols do this by utilizing the WNT signaling agonist CHIR. Next, for NP lineage derivation, posterior-intermediate mesoderm (PIM) must be induced, and for UB lineage, anterior-intermediate mesoderm (AIM) must be induced [17]. Interestingly, Takasato et al. (2015) found that the longer the period of CHIR administration, the more posterior-like mesoderm developed, while the shorter the period, the more anterior-like mesoderm developed [20]. Thus, increased WNT signaling duration leads to more glomerular and proximal structure generation, and decreased WNT signaling duration leads to more distal structure generation.

Upon NP protocol completion, organoids with both glomerular and tubular regions develop. However, they are immature. Studies have shown that hiPSC-derived kidney organoids mimic the first-trimester fetus [20]. One of the most extensive analyses on this topic was performed by Subramanian et al. (2019), who utilized RNA-seq to compare kidney organoids to 8-week, 17-week, and adult human kidneys. They concluded that kidney organoids are more similar to human kidneys in weeks 8 and 17 in the fetus than in adult kidneys [21]. Furthermore, kidney organoids show staining of primitive multipotent markers such as SIX2+ throughout the kidney, whereas the adult differentiated human kidney does not express such markers [22]. Therefore, to use the kidney organoid as a proxy for the adult human kidney to study disease, it must advance in gestational age.

Kidney organoids may not only more closely mimic early metanephros than the proper adult human kidney, but they may even more closely resemble the mesonephros than the metanephros if morphogen concentration is improperly regulated [23]. Addressing these concerns, Tsujimoto et al. (2020) investigated in vitro hiPSC differentiation into mesonephric NPs, metanephric NPs, and UB cells [24]. This study identified several factors that differentiate these three lineages and may, thus, be applied to the future study of these three distinct systems [24]. Other key advances in organoid maturation include NP-UB interactions and vascularization. Some of the most notable studies to address these issues were performed by Taguchi and Nishinakamura (2017) and Tsujimoto et al. (2020) [17,24]. These studies generated MM-UB co-cultured organoids and transplanted them into mice, where they were vascularized. Generation of these metanephric higher-order structures significantly advanced stem cell-derived kidney structure likeness to actual human kidneys. However, even these advanced systems were not able to recapitulate the more extensive UB branching that occurs throughout the second and final trimesters in vivo [24]. These findings underscore the need to replicate endogenous mature kidney functions and interactions that current organoids lack, such as fluid flow.

## 3. Kidney Organoids as Model Systems

The resemblance of kidney organoids to human kidneys makes them suitable for disease modeling and drug screening. They may be made in less than a month, personalized to an individual, and produced in bulk [12,13,14,17,18,19]. In addition, they may be cultured in vitro or transplanted into mice, rats, or chick eggs to form complete in vivo models. Below, we will explore analysis that may be conducted with kidney organoids, as well as current and future uses of kidney organoids in biomedical research.

### 3.1. Kidney Organoid Analysis

#### 3.1.1. In Vitro Assays

Various physiological, molecular, and functional assays may be performed on kidney organoids. In terms of molecular assays, different transcriptomic analyses have been successfully conducted in kidney organoids [25]. For example, Takasato et al. (2015) extracted RNA from organoids and performed RNA sequencing and qRTPCR analyses [20]. Others, such as Wu et al. (2018) have performed nuclei isolation and snRNA sequencing, in addition to DropSeq scRNA sequencing in kidney organoids. In addition to RNA levels, various protein levels have been quantified and compared from kidney organoid lysate via immunoblot (e.g., Cruz et al., 2017; Morais et al., 2022) [26,27].

**Figure 3:**
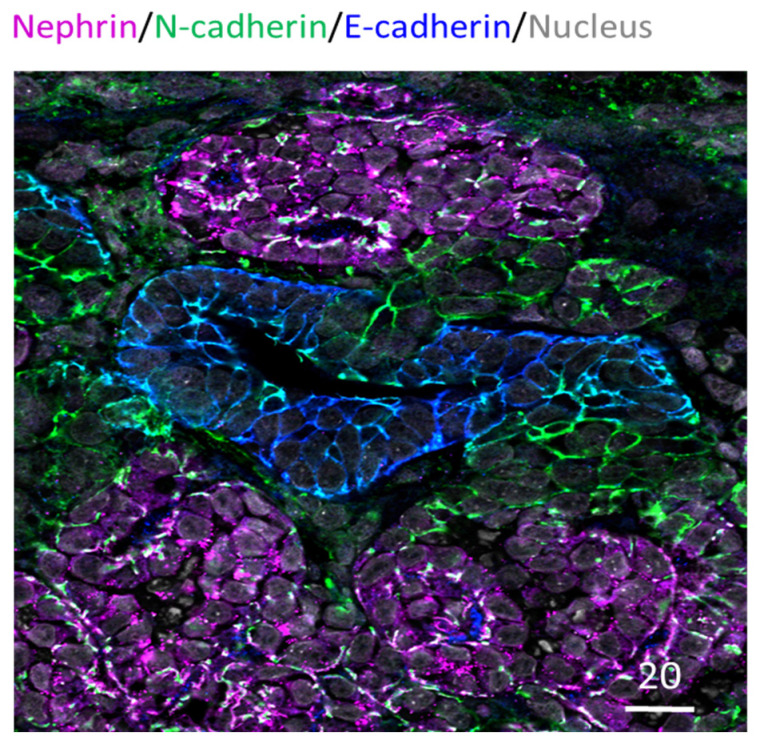
Human IPSC-derived kidney organoid. Kidney organoid section stained for glomeruli (Nephrin in magenta), proximal tubule (N-cadherin in green) and distal tubule (E-cadherin in blue) regions; also stained for nucleus in grey. Scale bar in microns.

Furthermore, immunocytochemistry analysis has been routinely performed in kidney organoids to examine specific nephron structures. This is often performed as whole organoid staining; alternatively, tissue sections have also been used for probing (e.g., Takasato et al., 2015; Cruz et al., 2017) [20,26]. These studies have revealed that kidney organoids exhibit glomeruli, proximal tubule, distal tubule, basal membrane, and collecting duct arrangement [20,27]. Figure 3 below shows an example of paraffin-embedded and sectioned human kidney organoids generated using the Takasato et al. (2016) protocol [12]. The section is stained for glomerular, proximal tubule, and distal tubule marker proteins and exhibits continuous glomerular to distal tubule nephronic structures. Besides these structures, the kidney organoids are known to exhibit vasculature as well. However, it is limited, quickly regressing and not organized as in a typical kidney [28].

Multiple functional assays may also be conducted in kidney organoids. For example, as described by Freedman et al. (2022), kidney organoids may be subject to pulse-chase assays, where various fluorescent molecules may be added to media before being replaced with new florescent-lacking media [29]. Organoids may then be analyzed for uptake of these molecules, and the resulting information can be used to deduce information on accumulation, swelling, filtration, endocrine, or injury [29]. However, one of the issues with this assay in organoid platforms is that molecules may be introduced externally to closed tubular structures instead of through the apical surface, as would occur in vivo. Hence, fluorescent molecules may be absorbed from the exterior basolateral membrane, as there is no way to control where these molecules may go when grossly introduced into the media. In addition, the transport of molecules can be limited by diffusion within organoids, creating different trends in accumulation within the same organoid.

Additionally, kidney repair may also be assessed in organoid platforms, thus allowing insights into kidney injury reversal mechanisms and genetic basis that may pre-dispose patients to kidney disease. For example, studies such as Gupta et al. (2022) have investigated gene pathways that were upregulated in the presence of a single or multiple exposures to cisplatin, a kidney injury molecule, in kidney organoid platforms [30]. Lastly, cyst formation may be analyzed in organoids for the purpose of studying polycystic kidney disease (PKD) via cAMP activation [26]. Cysts may then be measured, quantified, and treated as proxies for human kidney cysts.

#### 3.1.2. In Vivo Analysis

A significant drawback to using in vitro kidney organoids as a model system is their lack of interplay with the rest of the organism. A plethora of conditions that affect remote sections of the body can affect the kidney and vice versa. For example, changes in blood pressure can drastically change glomerular pressure, which the kidney can, in turn, regulate. However, in vitro organoid systems do not account for these systemic interactions. Thus, transplantation approaches that involve human-derived kidney organoids in mice, rats, and chick eggs have been explored. In such studies, human and animal tissues are distinguished via human nuclear antigen immunostaining or Y-chromosome read alignment to the combined genome reference [21]. Additionally, organoids can be transplanted via a scaffold (e.g., silk) to provide a higher level of structural integrity [31]. This approach may allow for easier organoid-tissue-specific analyses post-transplantation.

A key advantage of transplanting kidney organoids is that it allows organoids to vascularize, mature, and even filter urine. Van den Berg et al. (2018) have shown that after subcapsular renal transplantation into mice, kidney organoid glomeruli and tubules significantly mature [32]. In another study, Subramanian et al. (2019) have shown that transplantation of hiPSC-derived organoids into mice leads to increased proximal and distal tubule maturation and decreased presence of off-target cell populations within the organoid [21]. More importantly, post-transplantation, kidney organoids can perform the ultimate function of the kidney, filter blood. After subcutaneous transplantation in mice, kidney organoids formed urine-filtering structures, as evidenced by the transfer of FITC-labeled dextran [33]. Thus, not only does transplantation allow one to study the effects of a mutation in a kidney organoid in vivo, but it also improves the resemblance of the organoid to the human kidney and makes it a somewhat functional system.

While immunodeficient mice are often used for hiPSC kidney organoid transplantation, other hosts, such as chick chorioallantoic membranes (CAM), have also been used. A CAM is naturally immunodeficient and is conducive to vascularizing the organoid [16]. However, it lacks typical mammalian organ systems, making kidney organoids disconnected from other systems, unlike in mice. Despite host-related setbacks, chimera generation with human-derived kidney organoids allows for extensive in vivo study of human kidney disease in a highly impactful platform.

### 3.2. Disease Modeling Studies Conducted Thus Far

#### 3.2.1. Kidney Organoids as Genetic Disease Models

Kidney organoids have been used to study tubular and glomerular genetic kidney diseases [26,34,35]. In humans, the most prominent tubular diseases include pediatric polycystic kidney disease (PKD), which results from autosomal recessive mutations in the fibrocystin gene (PKHD1), and adult PKD, which results from autosomal dominant mutations in the polycystin-1 and -2 genes [36,37,38]. These mutations may be artificially introduced into hiPSCs via gene-editing technologies, such as the CRISPR-Cas 9 system. The edited hiPSCs may be subsequently grown into PKD-modeling human kidney organoids and analyzed via protein staining and RNA profiling. For example, Freedman et al. (2015) and Cruz et al. (2017) have knocked out polycystin genes in hiPSC lines using the CRISPR/Cas9 system and subsequently derived organoids that mimic the cystic phenotype found in vivo in diseased patients [14,26]. These studies show that kidney organoids may be used as easily observable, disease-relevant platforms to study PKD. Furthermore, organoids made from patient-derived cell lines allow insight into patient-specific mutations and the likelihood of disease development. For example, Low et al. (2019) developed kidney organoids derived from patients with PKHD1 mutations and compared them with wild-type organoids [39]. The diseased organoids exhibited significantly more cyst formation, thus demonstrating the potential of kidney organoids to predict disease manifestation from genotype [39]. In another study, Hernandez et al. (2021) derived hiPSCs from patients with mutations in the tuberous sclerosis complex-2 gene, which renders the patient prone to kidney tumor development [40]. They then corrected the patient mutation with the CRISPR/Cas9 system. Kidney organoids from these corrected isogenic mutant lines exhibited reduced cyst formation and restored gene pathways compared to the diseased organoids, thus demonstrating the usefulness of kidney organoids in studying the downstream functional effects of specific mutations in isogenic organ-like systems.

Researchers have also used patient-derived organoids to gain insight into genetic diseases that affect the glomerulus. For example, Hale et al. (2018) focused on the NPHS1 (nephrin) gene, which, if mutated, induces faulty podocyte foot process formation and leaky urine filtration, resulting in kidney disease [34]. Hale et al. (2018) used patient-derived mutant NPHS1 hiPSCs to derive kidney organoids that modeled these faulty podocyte foot processes, including decreased levels of podocyte-specific proteins, nephrin, and podocin [34]. Likewise, Freedman et al. (2015) knocked out the glomerular gene PODXL in hiPSCs and found that organoids show faulty podocyte-podocyte architecture [14].

One largely untapped potential of kidney organoids is their ability to model embryonic and fetal defects. Currently, researchers strive to model the adult human kidney with the kidney organoid. However, in its current first and second-trimester state, the kidney organoid may be used to study developmental kidney defects [41]. Urinary system developmental defects, such as kidney dysplasia, are among some of the most prevalent and severe developmental disorders [42]. Since many of these diseases may be detected as early as 11 weeks, kidney organoids lend themselves particularly well to studying congenital kidney disabilities. We not only have metanephric first and second trimester-like kidney organoids at our disposal, but we also have primitive mesonephric kidney cell lines (e.g., Tsujimoto et al., 2020) to study embryonic and fetal defects [24]. As few systems allow for the study and manipulation of a developing human kidney, the kidney organoid allows the tremendous potential for fetal genetic, toxicological, and developmental studies.

#### 3.2.2. Kidney Organoids as Models for Other Disease

In addition to genetic disease, kidney organoids have been used to study the response of human systems to viral infection, cancer, and injury. For example, Jansen et al. (2021) used a kidney organoid platform to evaluate the effects of viral SARS-CoV-2 on human kidneys [43]. This group discovered that SARS-CoV-2 infection leads to increased collagen-I expression in human kidney organoids, thus providing a mechanistic explanation of the kidney injury and fibrosis that often accompanies severe cases of long COVID-19 [43]. Regarding cancer research, Hernandez et al. (2021) transplanted kidney organoids into immunodeficient rats to model rare genetically influenced kidney tumors. Here, patient-derived organoids developed tumor-like lesions, thus recapitulating human kidney tumors [40]. In addition, they used kidney organoid-based tumor models as semi-functional human-derived structures and tested drugs and nanoparticle-based therapies. Finally, recent studies have shown that kidney organoids may be used to test kidney injury response. For example, Prezpiorski et al. (2022) demonstrated that kidney organoids produce markers of oxidative damage and increased injury marker expression by administering the injury molecule hemin to kidney organoids, along with a biosensor [44].

#### 3.2.3. Kidney Organoids in Drug Evaluation

**Figure 4:**
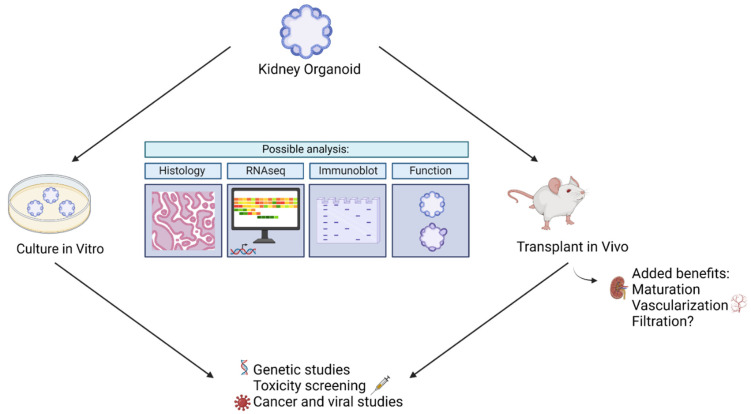
Summary of ways kidney organoids may be used to model disease.

In addition to providing insight into diseases, kidney organoids have been used to screen drugs. In particular, kidney organoids have been used to study drug-induced kidney injury (DIKI), which is a leading cause of acute kidney injury [45]. In the evaluation of cisplatin side effects, Czerniecki et al. (2018) showed that kidney organoids are helpful as high throughput models to vet new pharmaceuticals in a diverse array of human genetics [46]. They evaluated the toxic side effects of cisplatin via quantification of biomarkers, such as KIM-1, and quantification of apoptosis in kidney organoids derived from a variety of genotypes. They were able to show that certain genotypes were more likely to exhibit toxic side effects than others [46]. In addition, they established an automated kidney organoid derivation platform suited to toxicology assessment. Such assessments inform us whether a certain drug dosage can affect the kidney at a personalized level. Furthermore, these studies may administer drugs not only to the mature organoid, as was the case to study cyst development, but throughout the entire process to mimic in utero conditions. Thus, kidney organoids may aid us in gauging individualized reactions to drugs and in gauging gestational outcomes. Figure 4 below summarizes kidney organoid uses.

## 4. Strategies to Improve Kidney Organoid Platforms

Current kidney organoids exhibit kidney-relevant structures but do not resemble functional adult kidneys. Thus, the protocols described in Section 2.2 give rise to kidney organoids that lack an in vivo-like biophysical environment, including fluid flow through the tissues. Recent studies have improved organoid resemblance to the adult kidney, using techniques such as transplantation and combining different organoid types to achieve higher-order kidney structures. These techniques may be combined with bioengineering approaches to explore and improve organoid maturation. Below, we will highlight those strategies and discuss new considerations for generating next-generation kidney organoid systems.

### 4.1. Introducing Gradients

We know that during normal embryogenesis the mesonephros, metanephros, and UB send signals to one another to pattern the developing kidney [6]. In addition, biochemical gradients established in the early embryo are essential to directing proper kidney patterning and development [47]. A significant issue in our current kidney organoid development protocols is their isolation from other bodily tissues during development, and thus lack of biochemical gradients established via interactions with other co-developing tissue systems of an embryo. Therefore, it is worthwhile to include those interactions in organoid development. Taguchi and Nishinakamura (2017) and Tsujimoto et al. (2020) have considered some of these interactions to facilitate organoid maturation [17,24]. In their strategies, they first used PIM and AIM-specific factors to develop independent organoids, then transplanted co-cultured MM and UB organoids into mice [17,24]. Kidney organoids developed in this manner exhibited higher-order structures but could not recapitulate extensive in vivo-like UB branching. While these studies underscore the importance of co-culturing neighboring tissues, they call for the improvement of the organoid via established gradients included from the onset of organoid development.

As it is nearly impossible to include every tissue from the developing embryo and optimize its conditions, we propose a simulation of an environment, where the cells experience the effects of neighboring tissue. This may be achieved by engineering a biochemical gradient to grow cells, wherein the gradient can mimic the presence of neighboring tissues. The gradient may then direct both AIM and PIM lineages within the same suspension of cells, and thus induce UB and MM formation simultaneously. For example, we could establish a GDNF gradient and advance UB branching within the MM to induce the formation of the intricate collecting duct system of the adult kidney.

**Figure 5:**
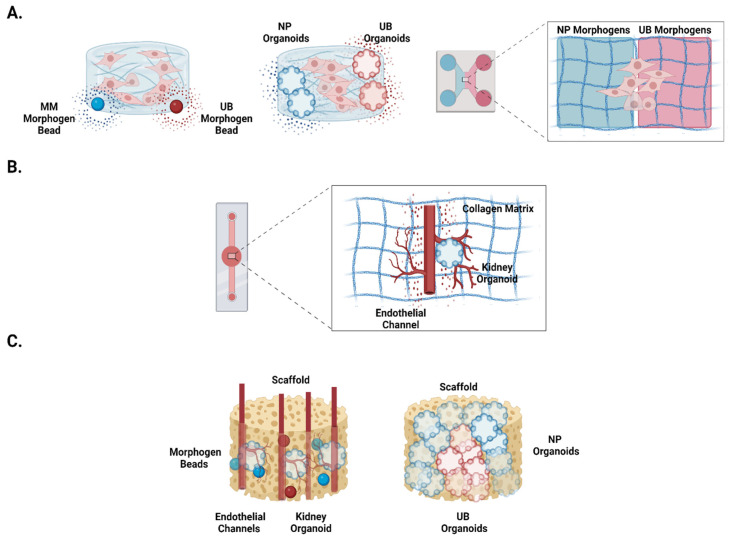
Proposed improvements to kidney organoid platforms. (**A**) Approaches to generate gradients in which hiPSCs may be cultured to evolve into kidney organoids. From left to right: iPSC cluster in hydrogel flanked by morphogen-emitting beads, hiPSC cluster in hydrogel flanked by morphogen-emitting organoids, multi-chambered chip system with hiPSC cluster cultured in a hydrogel and bilaterally exposed to morphogens of NP and UB lineage contained in a liquid. (**B**) Proposed chip system to culture a kidney organoid beside a branching endothelial channel, with flow through in a hydrogel (e.g., collagen matrix). (**C**) Proposed scaffolding set ups, in which kidney organoids can be grown and subsequently transplanted. From left to right: porous scaffold with branching endothelial channels (red), seeded kidney organoids, and seeded morphogen beads (red and blue spheres); porous scaffold with NP organoids surrounding UB organoids.

Although these gradient-based approaches have not been explored to establish kidney organoids, they have been used to improve the maturation of other organoids. For example, Ben-Reuven and Reiner et al. (2020) embedded morphogen-releasing beads in a hydrogel to help pattern the anterior–posterior axis of brain organoids [48]. In another study, Kamperman et al. (2019) established cell substratum with biotinylated morphogens [49]. Besides these static approaches, researchers have also developed microfluidic chips with flow-driven morphogen addition. For example, Cui et al. (2020) used a chamber with chemotaxis chambers for morphogen distribution and chambers into which stem cells may be cultured in hydrogels [50]. Thus, researchers may apply similar systems to program kidney organoid development using specific factors that are spatially controlled to mediate their effects. This would establish a gradient and mimic the naturally occurring morphogen distributions relevant to embryonic kidney development. For example, beads with factors specific to AIM and PIM differentiation may be seeded at opposite sides of a hydrogel to mimic natural AIM-PIM gradients. Alternatively, to control hiPSC differentiation, cells or organoids may be seeded into microfluidic devices, such that cells/organoids of different lineages are on opposite sides (see Figure 5A). HiPSCs embedded in hydrogels may be cultured in-between these two structures, which may serve as sources of morphogenic factors. This approach may be utilized to mimic interactions between the mesonephros and metanephros, which the current kidney organoid protocols lack in their setup (see Figure 5A). Thus, future work should aim to mimic biochemical cues from multiple tissue types that are relevant to kidney development, including those between the UB and MM, as well as between the developing kidney and vasculature. The related system design can explore the options of establishing gradients to mimic those interactions.

### 4.2. Perfusing Organoids with Microfluidics

Fluid flow is vital for kidney physiology, since it initiates a cascade of intracellular events in response to mechanosensing and facilitates transport across tissues [51]. Therefore, if we wish to generate functional kidney organoids and sustain them for extended periods of time, we must integrate stable and perfusable vascular networks into our organoid model systems. Fluid flow has been introduced to kidney organoids in two main ways, microfluidics and transplantation. Homan et al. (2019) included biophysical cues in a 3D bio-printed chip, wherein organoids were cultured in a chamber with dynamic fluid flow [52]. This setup simulated microfluidic flow and promoted vascular network formation, but it did not recapitulate perfusion through vascular and tubule structures to replicate kidney physiology. Nevertheless, this study highlighted the importance of the biophysical environment in organoid maturation.

We believe that one can develop organoids in a channeled microfluidic organ-on-chip platform to model the human kidney, as has been performed in other spheroidal systems. For example, unlike Homan et al. (2019), who placed an organoid in a fluid-flow chamber, Nashimoto et al. (2017) cultured human lung fibroblast spheroids in an extracellular matrix (ECM) between two endothelial channels [52,53]. The channels gave rise to a perfusable vascular network formed via angiogenesis. Using a somewhat similar approach to Nashimoto et al. (2017), we propose placing a hiPSC cluster next to a perfusable endothelial vessel that can be built into a chip, as shown in Figure 5B [53]. With the subsequent addition of sprouting kidney-organoid-specific signals, the vascular channel can be directed to branch and invade the organoid as it starts to differentiate. This configuration would replicate the early stages of kidney development [54]. With sustained perfusion of the necessary morphogenetic signals for kidney development, kidney organoids should be able to better replicate actual human kidney formation processes. It is also important that system parameters, such as fluid flow stress, ECM composition and configuration, and organoid stage are carefully considered and optimized. Furthermore, since Schumacher et al. (2021) have shown that hypoxia leads to increased angiogenesis in kidney organoids, such an ex vivo system may be cultured in the presence of decreased oxygen to more efficiently induce vascularization [55]. Ultimately, these strategies should facilitate organoid maturation, recapitulate vascular and tubular perfusion, and perform physiological functions as they occur in vivo.

### 4.3. Advancing Organoid Maturation via Transplantation

Studies have indicated that transplanting kidney organoids into a host, such as a mouse, chicken egg, or rat, may provide a viable route for vascularization [16,33,56]. However, transplanted organoids exhibit limited success as functional structures. A major hurdle in this strategy is recapitulating the extensive UB branching within the MM of transplanted organoids. Additionally, nephron segments within organoids lack proper orientation towards a collecting duct. These features are essential for the transplanted organoids to perform an adult human kidney’s function. Moreover, post-transplantation organoids integrate with the host, making it difficult to distinguish the organoid vs. host tissue boundaries, and thus limiting the organoid-specific analysis.

Tissue engineering strategies provide a wide range of tools to improve kidney organoid development in terms of maturation and functional integration. Typically, tissue engineering approaches employ scaffolds (e.g., silk fibroin scaffolds) in various configurations, such as films, mats, hydrogels, and sponges, to support the arrangement of cells and to recapitulate the different biophysical cues of the desired issues. For example, in one study, Gupta et al. (2019) used porous silk scaffolds to grow organoids, followed by transplantation [31]. In this approach, the scaffolding biomaterial and configuration provided a framework to support organoid development and conferred stability upon transplantation. However, this system lacked functional cues to support organoid maturation, such as the co-development of vasculature with the flow in proximity to other developing structures.

Future studies can develop similar strategies and include functional cues to support organoid maturation. For example, as outlined in Figure 5C, a porous scaffold can be configured with perfusable channels and loaded with morphogenic factors to develop a relevant organoid development system via transplantation. Pre-seeding with endothelial cells can support directed vessel development, whereas the open porous structure can be seeded with hiPSCs and programmed via morphogenetic factors for organoid development. Alternatively, the extracellular matrix (ECM) secreted from kidney tissues can, by itself, serve as a scaffolding material for tissue engineering a kidney organoid. For example, decellularized kidneys have been used to support the organoid’s maturation. Those matrices could be used to culture and transplant organoids or to derive tissue-engineering scaffolds from those biomaterials.

Furthermore, scaffolds present an untapped opportunity to embed multiple organoid lineages together in chosen positions. For example, Taguchi and Nishinakamura (2017) and Tsujimoto et al. (2020) transplanted both UB and NP organoids, allowing them to vascularize [17,24]; however, they lacked an extracellular scaffold to orient their multi-lineage organoids. By utilizing scaffolds, we could mimic nephron generation around a single collecting structure by orienting UB organoids in the center and bottom of a scaffold. At the same time, we can seed NP organoids along the exterior (see Figure 5C). Similar tissue engineering approaches can be developed to advance organoid maturation. Nevertheless, when designing a tissue engineering-based approach for transplanting the organoids, parameters such as biomaterials’ configuration and compatibility should all be carefully selected, as per the needs of the kidney.

## 5. Conclusions

The development of the kidney organoid is a major breakthrough in vitro kidney disease modeling. Kidney organoids allow for the individualized study of genetic kidney diseases and drug screening in a human-derived platform. While they hold tremendous promise, kidney organoids are immature and isolated in their current form. To truly model the human kidney and related diseases, we need to mature them by recapitulating developing kidney interactions. In particular, we need to advance the interactions between the UB and MM in vitro and introduce human-derived vasculature to kidney organoids. We must also consider gradient-based morphogen approaches to parallel in vivo human embryonic development. Kidney organoids hold great potential for genetic studies of adult and fetal kidney disease and drug screening. Their advancement may lead to the development of life-saving kidney disease treatments in a way that is faster and more accurate than ever before.
